# A monoamine oxidase B inhibitor ethyl ferulate suppresses microglia-mediated neuroinflammation and alleviates ischemic brain injury

**DOI:** 10.3389/fphar.2022.1004215

**Published:** 2022-10-13

**Authors:** Xinxin Zou, Shenghan Gao, Jiangnan Li, Chenggang Li, Chuyu Wu, Xiang Cao, Shengnan Xia, Pengfei Shao, Xinyu Bao, Haiyan Yang, Pinyi Liu, Yun Xu

**Affiliations:** ^1^ Department of Neurology, Nanjing Drum Tower Hospital Clinical College of Xuzhou Medical University, Nanjing, China; ^2^ Department of Neurology, Drum Tower Hospital, Medical School and the State Key Laboratory of Pharmaceutical Biotechnology, Institute of Translational Medicine for Brain Critical Diseases, Nanjing University, Nanjing, China; ^3^ State Key Laboratory of Natural Medicines, Jiangsu Key Laboratory of Drug Screening, China Pharmaceutical University, Nanjing, China; ^4^ Jiangsu Key Laboratory for Molecular Medicine, Medical School of Nanjing University, Nanjing, China; ^5^ Jiangsu Provincial Key Discipline of Neurology, Nanjing, China; ^6^ Nanjing Neurology Medical Center, Nanjing, China

**Keywords:** ethyl ferulate, ischemic stroke, microglia, neuroinflammation, monoamine oxidase B

## Abstract

Microglia are the resident macrophages in the brain, which play a critical role in post-stroke neuroinflammation. Accordingly, targeting neuroinflammation could be a promising strategy to improve ischemic stroke outcomes. Ethyl ferulate (EF) has been confirmed to possess anti-inflammatory properties in several disease models, including acute lung injury, retinal damage and diabetes-associated renal injury. However, the effects of EF on microglial activation and the resolution of post-stroke neuroinflammation remains unknown. Here, we found that EF suppressed pro-inflammatory response triggered by lipopolysaccharide (LPS) stimulation in primary microglia and BV2 cell lines, as well as post-stroke neuroinflammation in an *in vivo* transient middle cerebral artery occlusion (tMCAO) stroke model in C57BL/6 mice, consequently ameliorating ischemic brain injury. Furthermore, EF could directly bind and inhibit the activity of monoamine oxidase B (MAO-B) to reduce pro-inflammatory response. Taken together, our study identified a MAO-B inhibitor, Ethyl ferulate, as an active compound with promising potentials for suppressing post-stroke neuroinflammation.

## 1 Introduction

Ischemic stroke is considered to be one of the leading causes of death and disability worldwide that bring heavy burden to families and society. Previous evidence shows that neuroinflammation triggered within a few minutes after stroke, is a major contributor to ischemia-reperfusion brain injury and lasting neurological symptoms (Granger and Barnett, 2021; Jurcau and Simion, 2021).

Microglia, the innate immune cells in the cerebral nervous system acting, play a vital role in maintaining brain homeostasis for their diverse functions. Highly ramified resting microglia regulate brain development and neuronal network, eliminate synapse, and promote neurogenesis (Wake et al., 2013; Colonna and Butovsky, 2017; Wolf et al., 2017). As the sentinels in the brain, microglia could be active within minutes after ischemic insult and undergo significant morphological and transcriptional changes (Ma et al., 2017). The activation of microglia peak during the first week after ischemic stroke and persist for up to 30 days (Qin et al., 2019). Excessive activated microglia at the onset of ischemia sense the microenvironmental changes through diverse receptors including Toll-like receptors (TLR), Nod-like receptors and C-type lection receptors, thereafter enhancing the release of neurotoxic matters, such as superoxide, matrix metalloproteinases and several cytokines [e.g., interleukin-1β (IL-1β), interleukin-6 (IL-6), tumor necrosis factor-alpha (TNF-α)], which induces astrocyte activation, determines the fate of astrocyte (Jha et al., 2019) and aggravates ischemic brain injury. Additionally, activated microglia in the penumbra expended their protrusions toward blood vessels and engulf endothelial cells, which could disrupt blood-brain barrier (BBB) integrity (Jolivel et al., 2015). Accordingly, chemokines derived from microglia derive the infiltration of peripheral immune cells through the injured BBB, which further exaggerates neuroinflammation and worsens brain injury (Berchtold et al., 2020; Chen et al., 2021). Due to its detrimental role, microglia depletion at the early stage of experimental ischemic stroke suppressed the expressions of pro-inflammatory factors, resulting in neuroprotection (Li et al., 2021). For these reasons, the regulation of the neuroinflammation mediated by activated microglia is a promising target to rescue ischemic brain damage.

Ethyl ferulate (EF) is extracted from the medicinal herb Ferula and abundant in grains. A numerous studies suggest that EF exerts anti-inflammatory, antineoplastic, neuroprotective, antioxidative, anti-edema, antiproliferative effects on various disease models (Tsai et al., 2015; Cunha et al., 2019; Cunha et al., 2020; Kaikini et al., 2021; Kohno et al., 2020; Thapliyal et al., 2021). Recently, EF has been proved to own an anti-inflammatory effect on LPS-induced acute lung injury (Wu Y. X. et al., 2021), and protect neuron from Aβ1-42-induced oxidative stress in Alzheimer’s disease (AD) models (Sultana et al., 2005; Perluigi et al., 2006). Also, several evidence showed that EF inhibited the inflammatory response by AMPK/Nrf2, MAPK or NF-κB pathway (Islam et al., 2009; Wu Y. X. et al., 2021; Wang et al., 2022). However, the molecular target of EF still remains unclear.

Here, the objective of our study was to validate the neuroprotective effects of EF on ischemic stroke by alleviating microglia-mediated neuroinflammation. We found that EF significantly suppressed LPS-induced pro-inflammatory response and NF-κB pathway in primary microglia. Consistently, *in vivo* administration of EF also alleviated microglial activation and neuroinflammation, leading to attenuated ischemic brain injury. Intriguingly, EF was found to directly bind MAO-B and inhibit its activity, which contributed to the therapeutic effect of EF in microglia-mediated neuroinflammation.

Collectively, we found a MAO-B inhibitor ethyl ferulate with therapeutic potentials in neuroinflammation which was justified by both *in vivo* and *in vitro* experiments, providing a reliable neuroprotectant candidate for ischemic stroke.

## 2 Materials and methods

### 2.1 Materials

Ethyl ferulate (CAS:4046-02-0, Purity: ≥98%) was obtained from Yuanye Bio-Technology Co., Ltd. (Shanghai, China) and dissolved in Dimethyl sulfoxide (DMSO) for subsequent experiments. The concentration of DMSO in the culture medium was less than 1‰ (Kim and Lee, 2021). LPS (from *Escherichia coli* 0111: B4) and LDH Cytotoxicity Assay Kit were purchased from Sigma-Aldrich (St. Louis, MO, United States). Cell Counting kit-8 (CCK-8) was required from Dojindo laboratories (Tokyo, Japan). Antibodies against inducible nitric oxide synthase (iNOS), NF-kB (p65), Phospho-NF-kB (p-p65), Phospho-IκBα, IκBα, GFAP, TNF-α were purchased from Cell Signaling Biotechnology (Hertfordshire, United Kingdom). Antibodies against cyclooxygenase-2 (COX-2) and GAPDH were ordered from Bioworld Techonlogy (Shanghai, China). Recombinant Human Amine oxidase [flavin-containing] B (MAO-B) was purchased from CUSABIOM (Wuhan, China). Monoamine Oxidase inhibitor Rasagiline Mesylate (RM) was gained from Selleck (Shanghai, China). CheKine™ Monoamine Oxidase (MAO) Activity Colorimetric Assay Kit was got from Abbkine (Beijing, China).

### 2.2 Methods

#### 2.2.1 Animals

Nearly 8-week-old healthy C57BL/6 male mice for experiments were gained from GemPharmatch Co., Ltd. (Nanjing, Jiangsu, China), weighing from 22 g to 25 g. Experimental mice were housed in cages with standard dimensions, copious food and water, suitable light and temperature. All efforts were made to minimize pain and number of animals used.

#### 2.2.2 Cell culture and lipopolysaccharide treatment

BV2 microglia cells (Henn et al., 2009) were received from the China Infrastructure of Cell Line Resources (Beijing, China) and cultured in medium containing 90% MEM (Invitrogen, Frederick, MD, United States), 10% fetal bovine serum (FBS, Hyclone, Logan, UT, United States) and 1% antibiotics (100 U/ml penicillin and 100 μg/ml streptomycin) at 37°C in a humidified atmosphere of 5% CO2. Primary microglia were prepared from the cortical tissue of newborn C57/BL6J mice as reported earlier (Giulian and Baker, 1986). Ten to twelve days later, purified primary microglia cells in the suspension were collected by shaking the bottles for about 5 min and then seeded in 6-well or 12-well plates for following tests. The medium culturing primary microglia was all the same to BV2 cells other than DMEM. Microglial purification was confirmed greater than 95% by immunofluorescence staining with anti-Iba1. The concentration of LPS-stimulation on BV2 cells was 500 ng/ml while 100 ng/ml on primary microglia for subsequent experiments.

#### 2.2.3 Cell Counting kit-8 cell viability assay

The Cell Counting Kit-8 assay kit was used to assess cell survival. BV2 cells and primary microglia planted into 96-well plates were treated with different concentrations of EF (0, 5, 10, 20, 50 and 100 μmol) for 24 h. The medium was discarded and then CCK-8 solution was added into each well to incubate for another 2 h in the incubator. The absorbance at 450 nm was measured with a microplate reader. The value was calculated as a relative value.

#### 2.2.4 LDH cytotoxicity assay

The LDH cytotoxicity assay kit (Lu et al., 2018) was used to assess cell injury. The experiment was performed according to the manufacturer’s manual. In brief, cells were treated as above and then 40 μl of the medium supernatant was taken out to react with a same volume of LDH working solution in a new 96-well plate for 10 min at room temperature. The optical density (OD) reflected the concentration of LDH released by microglia in different groups was obtained at 492 nm with a microplate reader.

#### 2.2.5 Transient middle cerebral artery occlusion model establishment

The tMACO model, the most common model used in ischemia, was prepared as previously described (Meng et al., 2021). In a nutshell, after being anaesthetized with 2.5% Avertin (Sigma-Aldrich, United States; 100–200 μl/10 g *via* i.p. injection), 6/0 nylon sutures (Doccol Corporation, MA, United States) with a silicone heat-rounded tip were inserted into the MCA (middle cerebral artery) until the ipsilateral blood flow dropped below 30% of baseline level monitored using laser doppler flowmetry (Perimed Corporation, Stockholm, Sweden). For blood reperfusion, the filament was pulled out after 1 h occlusion. Sham-controlled groups were operated in accordance with tMCAO groups except filaments insertion. During the surgery, the body temperature of mice was maintained at 37 ± 0.5°C.

#### 2.2.6 Groups and drug administration

All experimental mice (N = 67, *n* = 12 per group for TTC staining and behavior tests (*n* = 8–9 for qRT-PCR or Western blot analysis), *n* = 6–7 for MAO activity detection and *n* = 3–4 for Immunofluorescence staining) were randomized to a sham-controlled group, a vehicle-treated (DMSO:1 × PBS (Phosphate Buffered Saline) = 1:20) tMCAO group and an EF-treated (EF:1 × PBS = 1:20) tMCAO group. Mice were subjected to tMCAO surgery except those in the sham group. After the surgery, the mice were returned to their earlier cages in a warm (25°C–26°C) environment with free access to food and water. Pre-dissolved 15 mg/kg EF or the same volume of vehicle was injected into the mouse peritoneal cavity subsequently at 30 min, 24 h and 48 h after tMCAO surgery and then returned to the cages mentioned above. All experiments were conducted in a double-blind manner.

BV2 cells and primary microglia cells were seeded into 6-well or 12-well plates for further experiments and divided into groups as following: control, DMSO-vehicle and EF with different concentrations (10, 20, 50 μmol). After vehicle or EF pretreatment for 2 h, all the cells excluded the control groups were exposed to LPS to induce inflammatory response.

#### 2.2.7 Nitric oxide detection

Primary microglia were replanted into 12-well plates and put in the incubator overnight. The next day, pre-treated with EF in different concentrations (10, 20 and 50 μmol) for 2 h and LPS (100 ng/ml) was added except control groups to induce microglial activation for another 24 h. The concentration of NO in the culture medium was detected using a Griess reaction kit (Beyotime Biotech, China) according to the protocol. The OD value reflected NO concentration was calculated at 540 nm with a microplate reader.

#### 2.2.8 Enzyme-linked immunosorbent assays

Primary microglia reseeded were treated as above. After being exposed with LPS for 24 h, the supernatants were collected. The protein levels of pro-inflammatory factors (IL-1β, IL-6 and TNF-α) were determined by enzyme-linked immunosorbent assays (ELISAs) according to the manufacturer’s instructions (Fcmacs Biotech, Nanjing, China). The OD value reflected the protein concentration of IL-1β, IL-6 and TNF-α in the supernatant was measured at 450 nm with a microplate reader.

#### 2.2.9 RNA isolation, cDNA conversion and quantitative RT-PCR

*In vitro*, BV2 cells and primary microglia seeded in 12-well plates were treated with EF for 2 h, prior to LPS-stimulation for another 3 h. *In vivo*, the mice subjected to tMCAO surgery were euthanized 3 days later with pentobarbital sodium (45 mg/kg i.p.) and put to death *via* cardiac perfusion with 1×PBS, then the tissues from ischemic-penumbra (right hemispheres) were extracted *via* 2% 2,3,5-triphenyltetrazolium chloride (TTC, Sigma-Aldrich) staining. Total RNA was isolated by using TRIzol reagent (Invitrogen) and reverse-transcribed into cDNA with PrimeScript RT Reagent Kit (Vazyme, Nanjing, China) according to the protocols. Preparing a 10ul reaction mixture before real-time PCR was performed using a LightCycle^®^ 96 Instrument Software system with SYBR Green Kit (Applied Biosystems). Primer sequences used were listed in Supplementary Table S1.

#### 2.2.10 Western blot

*In vitro*, followed by EF pre-treatment for 2 h, the grouped primary microglia were stimulated with LPS for 1.5 h or 24 h. *In vivo*, the ischemic penumbra tissues from grouped mice mentioned above were extracted on the third day of MCAO. Total proteins were extracted by using a pre-prep extraction solution consisting of lysis buffer (Thermo Fisher Scientific, Rockford, IL, United States) and 1% protease inhibitor, and concentrations were analyzed by a BSA kit (Beyotime). The protein samples mixed with 5 × loading buffer solution were separated with 10% SDS-PAGE gels and transferred to PVDF membranes. The membranes were blocked with 5% skim milk at 37°C for 1 h, and incubated with specific primary antibodies anti-iNOS (1:1000), anti-COX-2 (1:1000), anti-NF-kB (1:1000), anti-phospho-NF-kB (1:1000), anti-phospho-IκBα (1:1000), anti- IκBα (1:1000) and anti-GAPDH (1:5000) at 4°C overnight. The membranes were washed with 1 × TBST (3 times, 5–10 min each) and incubated with secondary antibodies. The protein bands on the membranes were visualized using a Gel-Pro system (Tanon Technologies, China). The integrated band densities were quantified *via* ImageJ software (ImageJ-win64, NIH, United States).

#### 2.2.11 Samples preparation and immunofluorescence

Primary microglia and coronal slices of mouse brains were pre-prepared for immunofluorescence staining according to a previous instruction (Liu et al., 2019; Zhang et al., 2020). Primary microglia cells were seeded in confocal petri dishes and divided into three groups: control, DMSO-vehicle and EF with a dose of 50 μmol. After been stimulated by LPS for 1.5 h, the cells were fixed with 4% paraformaldehyde for about 15 min. Grouped mice mentioned above euthanized with pentobarbital sodium were perfused with 1 × PBS and 4% paraformaldehyde *via* the left ventricle at day 3 after tMCAO. The brains were separated from the skulls carefully, then 20 μm coronal sections were sliced on a cryostat microtome (Leica, Wetzlar, Germany). Primary microglia and the brain tissue sections were pretreated with 0.25% Triton X-100 for 20 min and blocked with 2% BSA for additional 1 h. Then the samples were incubated in primary antibodies against goat anti-Iba1 (1:500), rat anti-Complement C3 (1:500, abcam), mouse anti-GFAP (1:300), rabbit anti-NF-kB (1:500) or mouse anti-TNF-α (1:500) overnight at 4°C. Then cells and sections were incubated with corresponding secondary antibodies in the dark at 37°C for 1.5 h the next day. Thereafter, DAPI (1:500) was added to constrain the nuclei for 15 min.

#### 2.2.12 Image processing, three-dimensional stacks of microglial morphology and skeleton analysis

Samples of primary microglia and brain sections were photographed using a fluorescence microscope (Olympus BX51, Japan) with 20X objective, or 40× amplification in zoom1 or zoom2 with an image matrix 1024 × 1024 pixel and a depth of 8 bit. In addition, representative three-dimensional (3D) reconstructional images of the morphology of microglia were analyzed by Imarsis software (Imarsis -win64, bitplane). Skeleton analysis was used as a tool to quantify microglial morphology. Briefly, confocal-images in a fixed area of brain sections were collected in Z-stacks with a scanning-laser of 1 μm after immunofluorescence then analyzed by using ImageJ skeleton analysis as previously described (Young and Morrison, 2018).

#### 2.2.13 Assessment of infarct size

Grouped mice mentioned as above were executed as above and brains were removed quickly 3 days after tMCAO surgery. Mouse brains were sliced into five sections and immersed into 2% TTC in order at room temperature 15 min for staining. The red area was the perfusion normal area, while the white area was the infarct area and the intersectional pink area was the penumbra. Slices were photographed with a camera and analyzed by ImageJ software. The value of infarct size was expressed as a percentage using the following formula: (all contralateral area—ipsilateral non-infarct area)/(2 × contralateral area) × 100% (Yang et al., 2020).

#### 2.2.14 Neurological assessments

All tests were conducted in a blinded manner. To assess the impairments of neurological function induced by cerebral ischemia, the modified neurological severity score (mNSS) test which integrated with motor (muscle status and abnormal movement), sensory (visual, tactile, and proprioceptive sensory) and reflex was introduced at the third day of tMCAO. Scores were graded from 0 to 18. The higher the score, the greater the damage was.

Experimental mice were trained twice a day on a rotarod device (RWD Life Science, Shenzhen, China) for 3 days before tMCAO-operation, during which the rotating rod was speed up from 10 to 40 rpm gradually, each training lasted for 5 min with a 15 min internal rest. All the mice moved steadily at 40 rpm on the last training session. On the third day after tMCAO, the time each mouse fell from the rod was recorded at the speed of 40 rpm.

Foot-fault test was used to evaluate the damage on the motor function. Mice were trained continuously for 3 days likewise. The numbers of foot faults within 50 total steps were counted *via* a video recording device on the day before and on the third day after tMCAO, respectively.

The forelimb muscle strength of the mice was assessed using the grip strength test. Before the test, mice needed to be trained once a day for 3 days. The test was performed as described below: each mouse was suspended by the tail, once grasping the platform of a grip strength metre (GS3, Bioseb, France) with two forelimbs, pulled backward in a straight line until its grip was broken. The average of grip force measurements was used as the muscle strength of forelimbs.

#### 2.2.15 Autodock and protein-ligand Interactional affinity analysis

Autodock, a computer virtual molecular docking technology (Meng et al., 2011), was used to explore the target of molecular ligands. Prediction progress on the interaction of protein-ligand was performed according to the tutorial. The affinity of Protein-ligand interaction was analyzed by surface plasmon resonance (SPR) technology, a label-free biophysical technology widely used in biomolecular interaction investigation including protein-ligand interaction (Tiwari et al., 2021), performing by BetterWays technology (Guangzhou, China).

#### 2.2.16 Monoamine oxidase-inhibition assay

The inhibitory effects of EF and/or Rasagiline Mesylate (RM, MAO-B inhibitor) on MAO-B were detected on treated BV2 cells and mouse ischemic cerebral hemispheres by the CheKine™ Monoamine Oxidase (MAO) Activity Colorimetric Assay Kit. BV2 cells used for all experiments were at a range from three to ten generations. *In vitro*, BV2 cells treated with EF were cultured with or without RM (50 μmol). Two hours later, LPS (500 ng/ml) was added except control groups for additional 3 h. Cells were washed with 1 × PBS and then harvested in Extraction buffer I. *In vitro*, grouped mice mentioned above (*n* = 6–7/group) were euthanized with pentobarbital sodium and then perfused with 1 × PBS transcardially. The infarcted lateral cerebral hemispheres (right hemispheres) were taken out. The weight of each brain sample (w) was recorded before soaked in the Extraction buffer I reagent. Further experimental steps were conducted strictly according to the instructions. The optical density (OD) value reflected the concentration of substrate (kynuramine, a nonselective substrate for MAO) was measured before and after substrate-protein interaction (difference showed as ΔA) at 360 nm with a microplate reader. MAO activity in BV2 cells was expressed by a relative value while an absolute value of tissue samples was calculated with a formula: 224 × ΔA/w.

#### 2.2.17 Statistical analysis

GraphPad Prism 8.0.2 software was used to analyze experimental data. Data were shown as mean ± Standard Error of Mean (SEM) derived from at least three independent experiments. Statistical significance between two groups was determined by Student’s t-test, while One-way ANOVA (analysis of variance) followed by Bonferroni’s post-hoc test was used for multiple comparisons among groups. Normality test was applied to the statistical analysis. A *p*-value <0.05 was regarded as statistically significant.

## 3 Results

### 3.1 Ethyl ferulate is brain-blood barrier permeable without observal cytotoxicity

**FIGURE 1:**
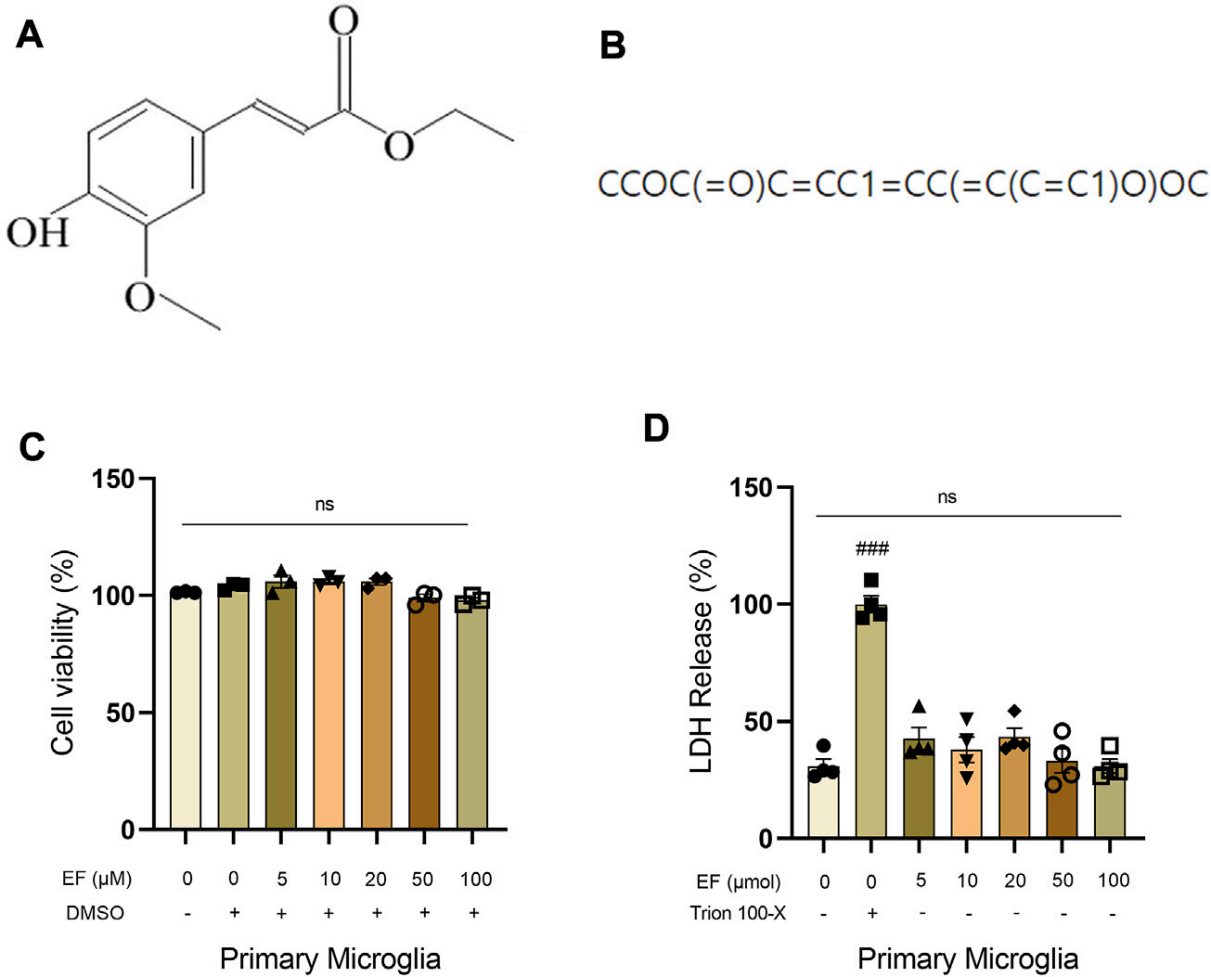
EF is blood-brain barrier permeable without observable cytotoxicity. **(A)** Chemical structure of EF. **(B)** Canonical SMILES computed by OEChem 2.3.0 (PubChem release 2021.05.07). **(C,D)** Primary microglia were treated with/without EF in a dose-dependent manner (0, 5, 10, 20, 50, 100 μmol) for 24 h, then CCK-8 assay and LDH cytotoxicity assay was added to detect cell viability, respectively. Values are presented as means ± SEM, *n* = 3–4/group. ###*p* < 0.0001 compared with control. ns represented no significant statistical difference. Comparison was from at least three independent experiments.

Ferulic acid (FA) is a compound with the ability to permeate the brain-blood barrier (BBB) (Stompor-Goracy and Machaczka, 2021). We speculated that EF is also BBB-permeable because it is a derivative from ferulic acid and has a hyperliposoluble phenylpropanoid structure (Figure 1A). As shown in Figure 1B, the Canonical SMILES of EF was computed by OEChem 2.3.0 (PubChem release 2021.05.07), through which we found that EF could pass the BBB easier than FA by using SwissADME website (Supplementary Table S2). Then, an CCK-8 assay and LDH cytotoxicity assay were performed to evaluate the possible cytotoxic effect of EF on primary microglia (Figures 1C,D). Primary microglia were treated with EF in a dose-dependent manner ranging from 0 to 100 μM for 24 h. Our data showed that EF did not produce any toxicity on the cells and was regarded to be safe even when the concentration reached 100 μmol. Considering that high-dose drugs still have limitations on clinical applications, the concentrations of EF we used in our subsequent experiments were below 100 μmol.

### 3.2 Ethyl ferulate suppresses lipopolysaccharide-induced proinflammatory response in primary microglia

**FIGURE 2:**
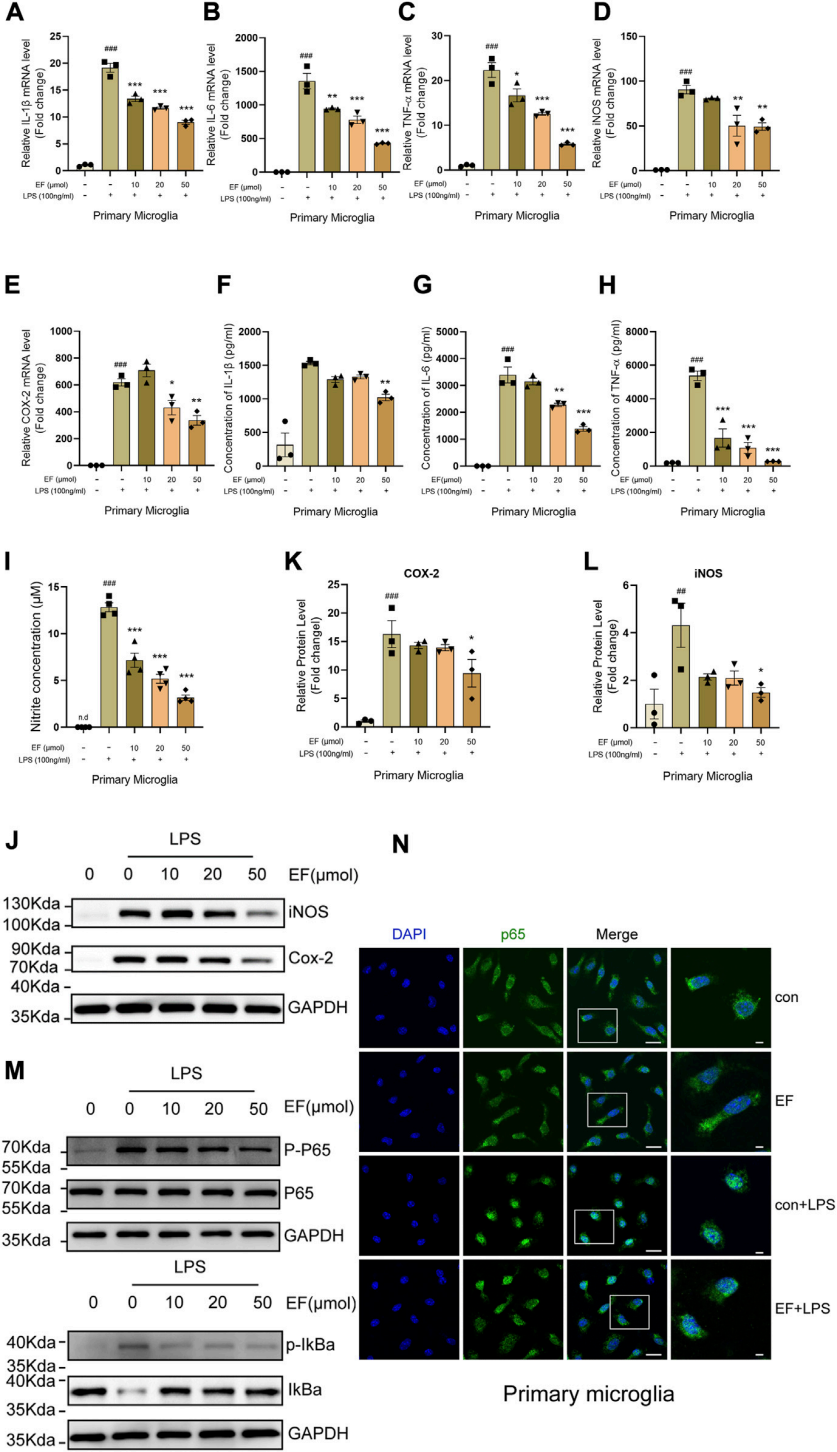
EF suppresses LPS-induced proinflammatory response in primary microglia. Primary microglia were treated with/without EF in different concentrations for 2h, prior to LPS (100 ng/ml) -stimulation. **(A–E)** The mRNA levels of IL-1β **(A)**, IL-6 **(B)**, TNF-α **(C)**, iNOS **(D)** and COX-2 **(E)** were quantified by RT-qPCR. **(F–I)** The protein levels of IL-1β **(F)**, IL-6 **(G)** and TNF-α **(H)** were detected using ELISAs and the concentrations of NO **(I)** in the supernatant were tested using the Griess reaction kit. **(J)** The protein levels of iNOS and COX-2 in cell lysates were measured by western blot with GAPDH as a loading control. **(K,L)** The ratio of COX-2/GAPDH **(K)** and iNOS/GAPDH **(L)** was analyzed by ImageJ software. **(M)** The protein level of p-p65, p65, p-IkBα, IkBα and GAPDH were detected using western blot. **(N)** The treated primary microglial cells were stained with NF-κB p65 (green) and DAPI (blue). Immunofluorescence microscopy was used to observe the localization of NF-κB and nuclei, right images represented amplified images. Scale bars = 100 μm and 50 μm. The values are shown as the mean ± SEM, *n* = 3–4/group. ##*p* < 0.005, ###*p* < 0.001 compared with control groups. *p < 0.05, **p < 0.005, ***p < 0.0001 compared with LPS-treated groups. N.d represented none-detection.

Given that EF was previously identified to be an anti-inflammatory compound, we hypothesized that EF could suppress microglia-mediated neuroinflammation. LPS, a well-characterized TLR4 agonist was used to activate primary microglia. After LPS-stimulation, total RNA, culture medium and protein samples were collected for subsequent detections (RT-qPCR, ELISA and Western blot assessments). The results showed that EF-treatment suppressed the expression of IL-1β, IL-6, TNF-α, iNOS and COX-2 both at mRNA and protein levels, and reduced the concentration of NO in the cell supernatant induced by LPS in a dose-dependent manner (Figure 2A–L). Neuclear factor kappa-B (NF-κB), a canonical regulator of inflammation and immunity, is activated by inflammatory stimuli and translocated into the nucleus to trigger the transcription of downstream proinflammatory genes, involving in various cellular functions (Guo et al., 2022; Moore et al., 2022). As shown in Figures 2M,N, EF-treatment could reduce the ratio of phosphorylated-NF-κB p65/p65, phosphorylated-IκBα/IκBα and interfere the translocation of NF-κB p65 from cytoplasm to nucleus in LPS-stimulated primary microglia. Thus, our data showed that EF was effective in suppressing proinflammatory response triggered by LPS-stimulation in primary microglia. To ensure the effectiveness of our experiments, we verified the anti-inflammatory effect of EF on LPS-induced BV2 cells at first (Supplementary Figure S1).

### 3.3 Ethyl ferulate alleviates microglial activation and inhibits post-stroke neuroinflammation after ischemia

**FIGURE 3:**
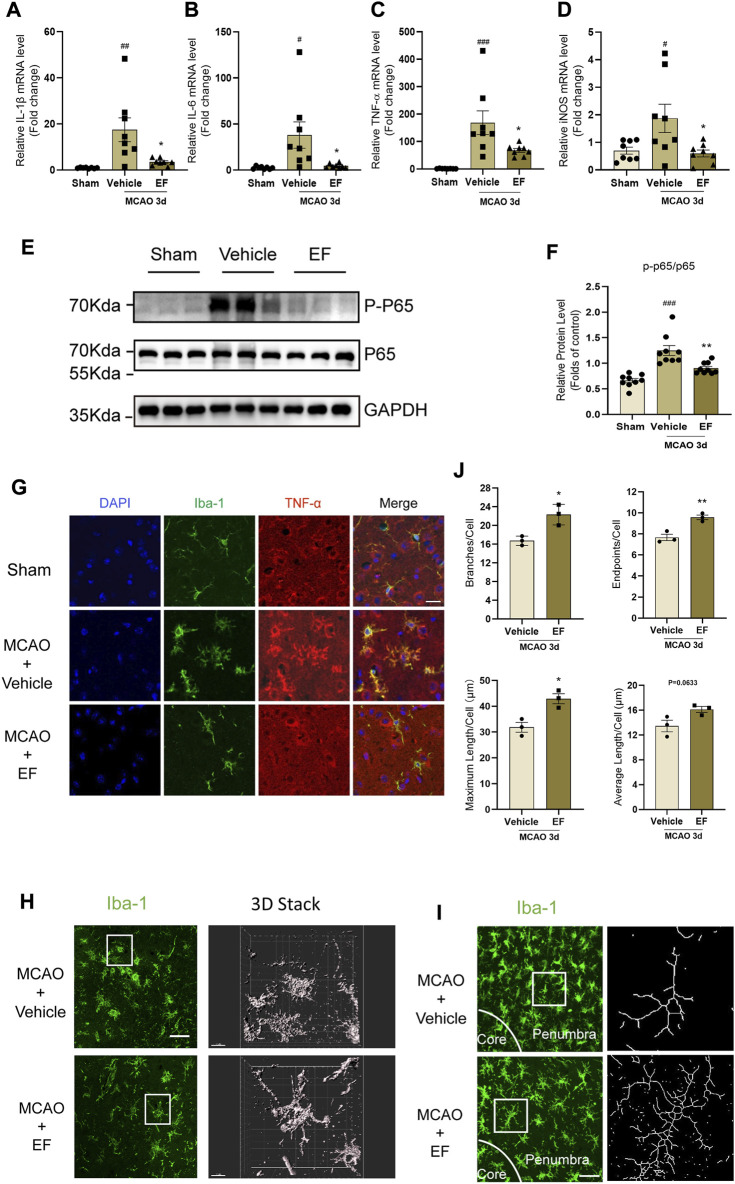
EF alleviates microglial activation and post-stroke neuroinflammation after ischemia. Mice subjected to tMCAO were divided into three groups, the MCAO groups were administered with vehicle or EF *via* i.p. injection for 3 days. Then the ischemic penumbra of brain tissue from grouped mice was collected for RT-qPCR or western blot. **(A–D)** Total RNA of penumbra tissue was extracted and the mRNA levels of IL-1β, IL-6, TNF-α and iNOS were assessed by RT-qPCR (*n* = 8/group). **(E,F)** P-P65, P65 and GAPDH protein levels of homogenate tissue were detected by western blot. The quantifications of relative band intensities were determined by densitometry using ImageJ software (*n* = 9/group). **(G)** Brain tissue slices from grouped mice were stained with Iba-1 (green), TNF-α (red) and DAPI (blue). Scale bars = 20 μm. **(H)** Images left staining with Iba-1 (Green) were obtained using Immunofluorescence microscopy, and three-dimension reconstructional images of microglial morphology right were analyzed by Imaris software (Scale bars = 50 μm and 10 μm, respectively). **(I)** Confocal images with corresponding binary of microglia on brain sections in EF-treated and controlled mice 3 days after MCAO, labeled with Iba1 (green). Scale bar = 20 μm. **(J)** Microglial branches/cell, endpoints/cell, maximum length/cell and average length/cell in vehicle and EF treated mice after ischemia were calculated by using ImageJ, *n* = 3/group. Values are expressed as the mean ± SEM. #*p* < 0.05, ###*p* < 0.005 versus sham groups. *p < 0.05, **p < 0.005 compared with vehicle-treated groups.

Neuroinflammation is one of the hallmarks of post-stroke pathophysiology, and microglia plays a pivotal role in this process. To investigate the therapeutic effect of EF on ischemic stroke, mice subjected to tMCAO were intraperitoneally injected with EF or not. Our results showed that the mRNA levels of IL-1β, IL-6, TNF-α and iNOS in the penumbra at the third day after tMCAO were downregulation after EF administration (Figures 3A–D). Consistent with our results *in vitro*, the protein level of phosphorylated-NF-κB p65 in EF-treated mice was lower as compared with the vehicle-treated mice (Figures 3E,F). Furthermore, TNF-α was co-stained with Iba1 to illustrate the association between neuroinflammation and activated microglia (Figure 3G). Indeed, EF administration could decrease the expression of TNF-α in microglia and remarkably switched the morphology of microglia from an ‘amoeboid’ state to a relative ‘resting-like’ state with smaller cell bodies, more branches and endpoints, longer maximum and average length (Figures 3H–J), indicating EF an inhibitory effect on microglial activation. Additionally, neuroinflammatory microglia could induce A1 reactive astrocytes to aggravate the brain injury. GFAP and complement component C3 (a marker of A1 reactive astrocytes) were used to co-label reactive astrocytes. It was found that EF administration could reduce the percentage of C3 positive GFAP astrocytes in the penumbra tissue of mice compared with the those treated with vehicle (Supplementary Figures S3A,B). Taken together, our results indicated that EF administration alleviated the activation of microglia, as well as post-stroke neuroinflammation after ischemia, highlighting the anti-inflammatory effect of EF in *in vivo* microenvironment.

### 3.4 Ethyl ferulate reduces infarct size and ameliorates neurological deficits in experimental stroke

**FIGURE 4:**
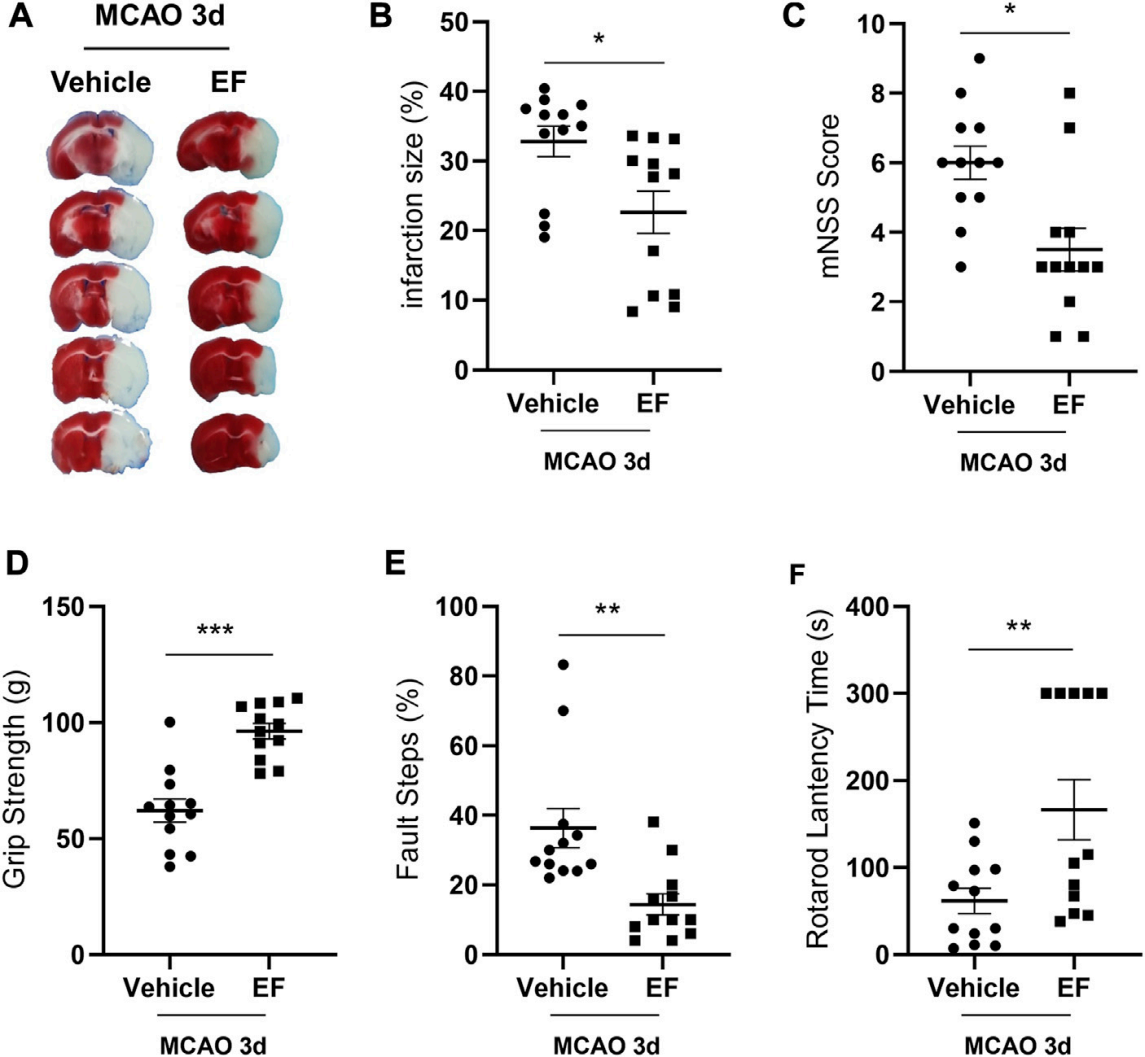
EF reduces infarct size and ameliorates neurological deficits in experimental stroke. Mice subjected to MCAO were administered with vehicle or EF (15 mg/Kg, once a day for 3 days) *via* i.p. injection. **(A)** Representative images of brain sections stained with TTC at the third day after tMCAO. **(B)** Infarction volume. **(C)** The mNSS scores. **(D)** The results of grip strength. **(E)** The results of fault steps. **(F)** The results of the rotarod test. Values shown are expressed as the mean ± SEM, *n* = 12/group. *p < 0.05, **p < 0.005, ***p < 0.0001 compared with vehicle-treated MCAO group.

Since neuroinflammation is one of the determinants of stroke outcomes, we investigated that whether EF may ameliorate ischemic brain injury. The results of TTC showed the infarct size of the EF-treated group was smaller than that of the vehicle group after tMCAO (Figures 4A,B). In addition, neurological defects in EF-treated groups were significantly attenuated as compared with the vehicle group as indicated by lower mNSS scores (Figure 4C), enhanced grip strength (Figure 4D), fewer fault steps (Figure 4E) and prolonged rotarod lantency time (Figure 4F), suggesting EF was a promising neuroprotectant in the treatment for ischemic stroke.

### 3.5 Ethyl ferulate suppresses pro-inflammatory response by targeting monoamine oxidase-B

**FIGURE 5:**
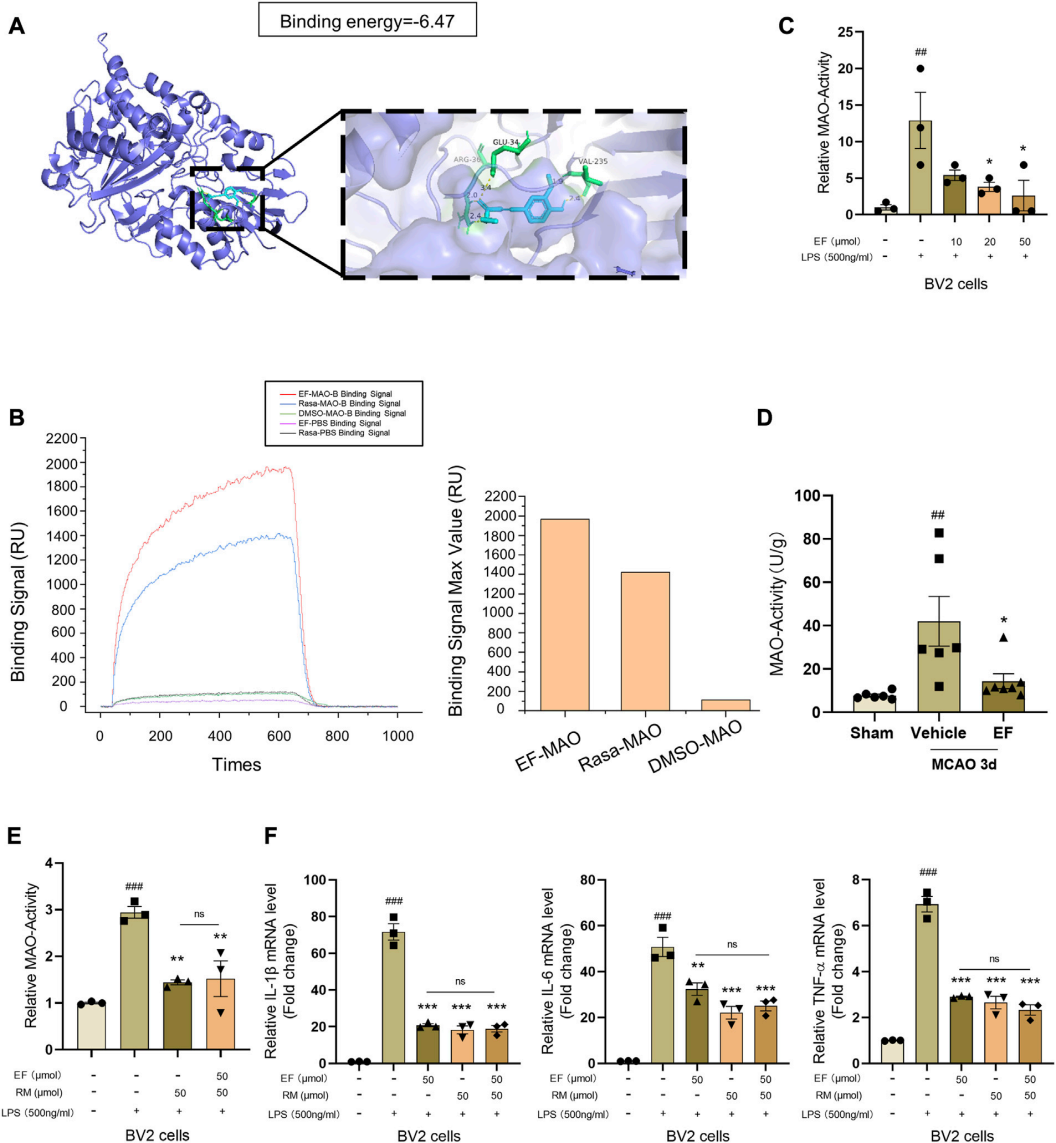
EF suppresses pro-inflammatory response by targeting MAO-B. **(A)** Crystal structures and binding energy of MAO-B in complex with EF predicted by Autodock software. **(B)** Protein (hMAO-B)-ligand (EF/RM) interaction and affinity analysis were conducted by Surface Plasmon Resonance (SPR), DMSO was used as a negative control. **(C–E)** BV2 cells were treated with/without EF or RM for 2 h, then stimulated with LPS (500 ng/ml) for additional 3 h. MAO-activity of treated cells (*n* = 3/group) and infarcted lateral hemisphere homogenate from grouped mice (*n* = 6–7/group) as above were tested using MAO Activity Colorimetric Assay Kit. **(F)** Total mRNA of treated BV2 cells was isolated and the mRNA levels of IL-1β, IL-6 and TNF-α were analyzed using RT-qPCR. Values are shown as the mean ± SEM. ##*p* < 0.005, ###*p* < 0.001 compared with control groups. **p < 0.005, ***p < 0.0001 compared with LPS-treated groups. ns > 0.05 represented no difference with each other.

In order to clarify the molecular mechanism underlying the anti-inflammatory effect of EF, SwissTargetPrediction was to predict the potential target of EF, and we found monoamine Oxidase-B (MAO-B) gained the highest score in both human and mouse proteins (Supplementary Table S3). Two isoforms of MAO encoded by *MAOA* and *MAOB* genes belong to the family of flavin-containing amine oxidoreductases, showing functional differences for their anatomic localization and major substrate (Fisar, 2016; Shahid Nadeem et al., 2022). Previous studies have found that the inhibition of MAO activity can modulate LPS-induced microglial activation *in vitro*, suppressing the expression of pro-inflammatory factors (Obuchowicz et al., 2006; Dhami et al., 2013; Park et al., 2020; Mariani et al., 2022). Rasagiline Mesylate (RM), a well-known irreversible MAO-B inhibitor, could suppress the expression of pro-inflammatory cytokines in LPS-treated BV2 cells (Supplementary Figure S2). We used AutoDock to simulate the drug-protein interaction and found that EF could bind to the active site (substrate-binding site) of human MAO-B (hMAO-B) by hydrogen bound in a relative low binding energy (Binding energy = -6.47 kcal/mol, Figure 5A). Furthermore, SPR analysis showed that EF exhibited a higher affinity with hMAO-B (Figure 5B) than RM, suggesting hMAO-B was a molecular target of EF. Our further data showed that MAO-activity were upregulated in LPS-activated BV2 cells and the penumbra tissues at 3 days after tMCAO, which however could be inhibited by EF-treatment (Figures 5C,D). In addition, EF/RM co-treatment did not show additive effect in MAO-activity (Figure 5E) and LPS-induced pro-inflammatory effect (Figure 5F), indicating that EF suppressed microglia-mediated neuroinflammation in a MAO-dependent manner. In summary, our study identified a novel MAO-B inhibitor, ethyl ferulate, which could suppress pro-inflammatory effect in activated microglia and alleviate ischemic brain injury, emerging as a promising candidate for the development of neuroprotectants in the treatment of ischemic stroke.

**FIGURE 6:**
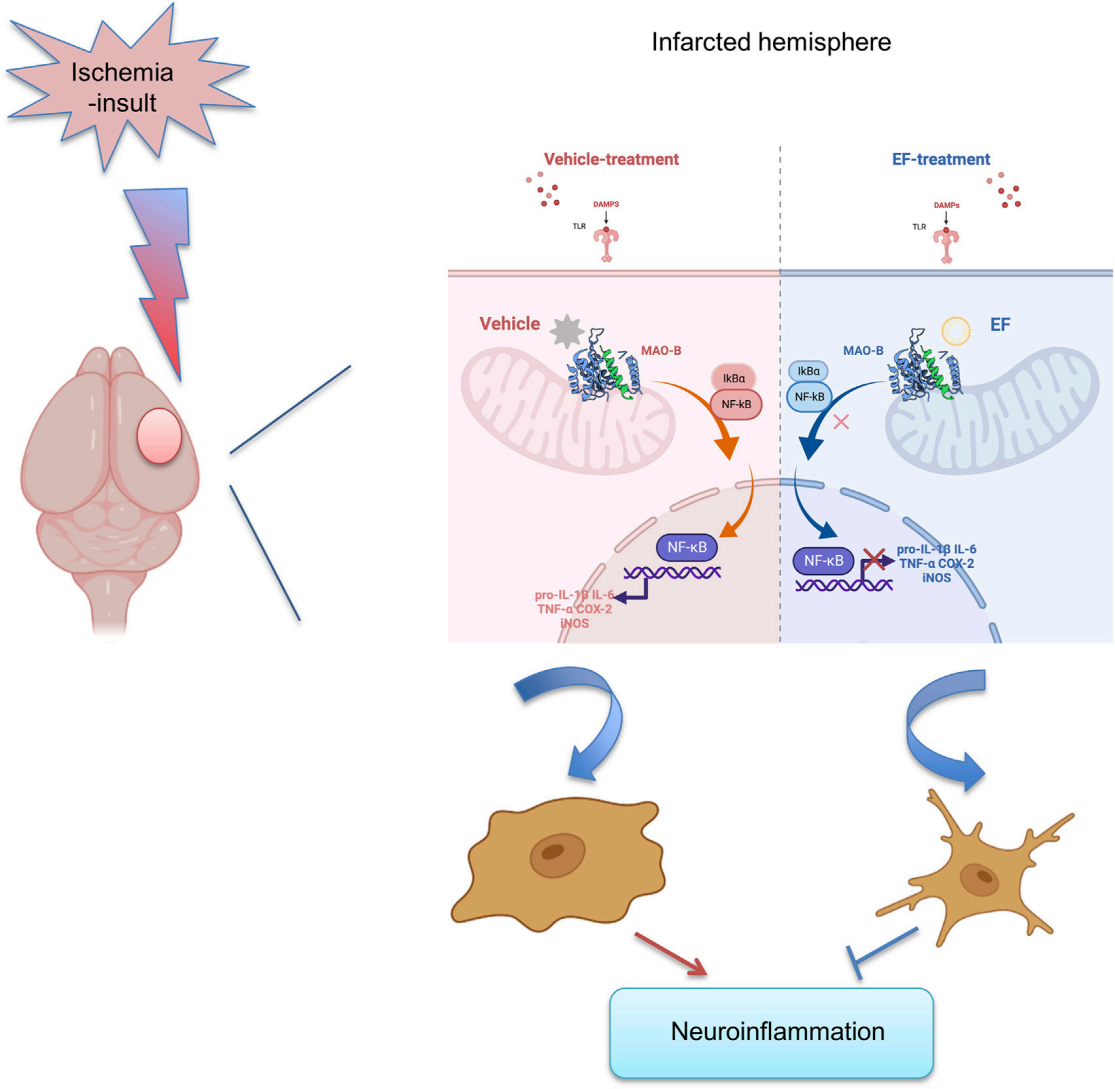
Pattern diagram of EF in suppressing neuroinflammation by alleviating microglial activation, reducing the expression of pro-inflammatory mediators including IL-1β, IL-6, TNF-α, COX-2 and iNOS *via* inhibiting MAO-B activity after ischemic stroke.

Collectively, EF-treatment could suppress neuroinflammation by alleviating microglial activation *via* inhibiting MAO-B activity after brain ischemia, providing a novel and promising therapeutic agent for ischemic stroke (Figure 6).

## 4 Discussion

Post-stroke pathophysiology is very complicated and has not completely understood, bringing difficulties to the development of therapeutics strategies. Notably, acute neuroinflammation caused by microglia hyperactivation contributes to poor outcomes in ischemic stroke (Petrovic-Djergovic et al., 2016). Mounting evidence suggest that activated microglia migrate to damaged regions and initiate a profound pro-inflammatory response by secreting cytokines and chemokines at the acute phase of ischemia (Cisbani et al., 2018; Candelario-Jalil et al., 2022), increasing the BBB permeability, neuronal injury and the infiltration of peripheral immune cells to aggravate neurological outcomes (Dudvarski Stankovic et al., 2016; Shaheryar et al., 2021; Mao et al., 2022). Therefore, targeting microglial activation and neuroinflammation might be a promising approach to ameliorate ischemic brain injury after stroke. So far, both the effects and the targets of EF in ischemic stroke have not been investigated, and our study demonstrated that EF could potently suppress microglia-mediated neuroinflammation by binding to MAO-B after ischemia, presenting a potential therapeutic compound in the treatment for ischemic stroke.

Pro-inflammatory cytokines are considered to exert deleterious roles after ischemic stroke. It was reported that activated microglia are the main sources of several cytokines (e.g., TNF-α and IL-1β) during ischemia, especially at the acute phase (Greenway and Dolman, 1999; Clausen et al., 2008), and minocycline, a commonly used agent to suppress microglial activation, could reduce infarct volume, preserve BBB integrity, and promote functional recovery in experimental stroke (Yang et al., 2015; Lu et al., 2021). In addition, inhibition of TNF-α, a strong pro-inflammatory cytokine that increases neuronal death and BBB damage, exhibit neuroprotective effect in rodent models of ischemic stroke (Sumbria et al., 2012; Sumbria et al., 2013; Arango-Davila et al., 2015). Furthermore, the crosstalk between microglia and astrocyte is crucial for brain development, homeostasis and diseases (Vainchtein and Molofsky, 2020). A previous study suggested that activated neuroinflammatory microglia induced A1 reactive astrocytes by secreting IL-1, TNF-α and C1q, which were considered as best inducers of astrocyte A1 phenotype in both *in vivo* and *vitro* experiments (Liddelow et al., 2017). In addition, complement component C3 was considered as a marker of A1-astrocyte since it is the most characteristic and highly upregulated gene in A1 reactive astrocytes (Gharagozloo et al., 2021). Reactive A1 astrocyte induced by activated microglia caused complement cascade reaction, synapses engulfment, neuronal loss and gliosis, aggravating brain injury and hindering brain functional recovery after stroke (Shi et al., 2021). In this study, we used LPS stimulation to activate *in vitro* cultured primary microglia and BV2 cells and found that EF could suppress LPS-induced pro-inflammatory response. Consistently, EF showed strong anti-inflammatory effect in MCAO mice as it remarkably harnessed pro-inflammatory response and reactive A1 astrocytes after ischemia. Specifically, microglia also showed a relative steady morphology and lower level of TNF-α expression after EF administration after MCAO, indicating that EF might attenuate ischemic brain injury *via* suppressing microglia-mediated neuroinflammation.

NF-κB pathway is a canonical signaling in pro-inflammatory response, as well as microglial activation in neuropsychological diseases (Frakes et al., 2014; Dutta et al., 2021). Under inflammatory stimuli, NF-κB p65 is phosphorylated and translocated to the nucleus, initiating the transcription pro-inflammatory genes (Yu et al., 2020). Our study found that EF treatment significantly reduced the ratio of phosphorylated-p65/p65 in both *in vitro* and *in vivo* studies, suggesting an inhibitory effect of NF-κB pathway might be involved in the therapeutic effect of EF on ischemic stroke.

MAO-B, a metabolic enzyme for accounting for 80% of total MAO in the brain (Ramsay, 2016) while only a little existed in peripheral platelets and lymphocytes (Naoi et al., 2016), is considered to be expressed in neurons and glial cells. MAO-B is associated with neuronal apoptosis, cellular excitotoxicity and oxidative stress in neuropsychiatric diseases such as Parkinson’s disease (PD), Alzheimer’s disease (AD) and depression (Tong et al., 2017; Park et al., 2019; Wu X. et al., 2021). It has been recently reported that ROS produced by MAO-B caused mitochondrial dysfunction and NF-κB activation, leading to NLRP3 inflammasome activation and IL-1β overexpression (Sanchez-Rodriguez et al., 2021). MAO inhibitors have been reported to reduce the expression of pro-inflammatory factors and attenuate inflammation, such as ischemia/reperfusion tissue injury, Parkinson’s disease, and smoke-induced lung damage (Sturza et al., 2019; Liu Y. et al., 2020; Ostadkarampour and Putnins, 2021). Here, our study found that EF could directly bind to MAO-B and inhibit its activity, indicating that the MAO-B inhibition might contribute to the anti-inflammatory and neuroprotective effects of EF on ischemic stroke. A recent study also discovered several highly selective MAO-B inhibitors and validated their anti-inflammatory effects in microglia, further supporting that MAO-B is a promising therapeutic target in neuroinflammation (Hassan et al., 2022). Additionally, given that MAO-B is also a therapeutic target in Parkinson’s disease (PD), we proposed that EF might have potentials as a MAO-B inhibitor to treat PD, which needs further validation in preclinical rodent models of PD.

However, there were still several limitations existing on our study. First, leukocytes including neutrophils, monocytes/macrophages and lymphocytes could infiltrate the ischemia brain (Jian et al., 2019; Kim and Cho, 2021), leading to brain edema and further aggravation, and our experiments could not completely rule out the effect of EF on peripheral immune cells. Dendrimers acted as a nanomaterial can be maneuvered to transport diverse therapeutic agents, diminish their cytotoxicity and improve their efficacy, showing a great potential for a noninvasive and accurate treatment (Zhu et al., 2019) and we hope that we can apply this technique to subsequent experiments in the future. Second, we administrated EF intraperitoneally in a fixed concentration without assessing optimal dosage, pharmacokinetics, and toxicity. In addition, we did not compare the efficacy between EF and rasagiline in experimental stroke, thus we cannot show the superiority of EF. Actually, MAO-B is related to dopamine metabolism in the brain dopaminergic neuron, and the inhibition of MAO-B can increase the level of dopamine *in vivo*. A “Dopamine Augmented Rehabilitation in Stroke (DARS) trial” on stroke patients also suggested that an elevated dopamine level was beneficial to stroke outcomes (Bhakta et al., 2014). However, our study did not evaluate the effect of EF on dopamine production. In addition, since we did not treat *Maob*-knockout mice with EF after stroke, we could not confirm that the neuroprotective effect of EF was completely dependent on MAO-B inhibition. Furthermore, it should be remembered that EF in our study suppressed inflammation and NF-κB signaling pathway through inhibiting MAO-B, but detailed mechanism linking MAO-B activity to microglia-mediated neuroinflammation remains elusive. We only speculated that MAO-B inhibition might reduce the expression of CREB (cAMP-response element binding protein) (Xia et al., 2015) and PPARγ/SIRT1 (Morsy et al., 2022) to suppress NF-κB signaling pathway according to previous study. In conclusion, we discovered a MAO-B inhibitor, ethyl ferulate, which shows a strong neuroprotective effect by targeting microglia-mediated neuroinflammation after ischemia, providing a candidate for the treatment of ischemic stroke.

## Data Availability

The original contributions presented in the study are included in the article/Supplementary Material, further inquiries can be directed to the corresponding authors.
