# Supplementary material for: A monoamine oxidase B inhibitor ethyl ferulate suppresses microglia-mediated neuroinflammation and alleviates ischemic brain injury
Source: Front Pharmacol. 2022 Oct 13;13:1004215. doi: 10.3389/fphar.2022.1004215 (PMC9608666; doi:10.3389/fphar.2022.1004215)
Supplement: Supplementary file 1 [file DataSheet1.PDF]

SUPPLEMENTARY MATERIAL

Gene		Sequence
GAPDH	Forward	5'-AGGTCGGTGTGAACGGATTTG-3'
	Reverse	5'-TGTAGACCATGTAGTTGAGGTCA-3'
IL-1 β	Forward	5'-CCATCCTCTGTGACTCATGGG-3'
	Reverse	5'-TCAGCTCATATGGGTCCGAC-3'
IL-6	Forward	5'-GACAAAGCCAGAGTCCTTCAGAGAG-3'
	Reverse	5'-CTAGGTTTGCCGAGTAGATCTC-3'
TNF- α	Forward	5'-CCACCACGCTCTTCTGTCTA-3'
	Reverse	5'-GATCTGAGTGTGAGGGTCTGG-3'
COX-2	Forward	5'-AGGACTCTGCTCACGAAGGA-3'
	Reverse	5'-TGACATGGATTGGAACAGCA-3'
iNOS	Forward	5'-GTTCTCAGCCCAACAATACAAGA-3'
	Reverse	5'-GTGGACGGGTCGATGTCAC-3'

Supplementary Table S1| Primer Sequences used in RT-qPCR. Primer sequences such GAPDH, IL-1β, IL-6, TNF-α, COX-2 and iNOS used for RT-PCR analysis were listed in the table.

Molecule 1

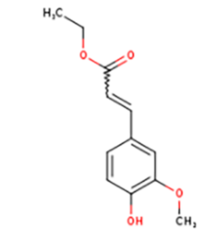

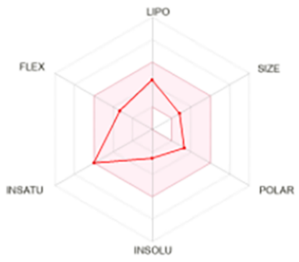

SMILES CCOC(=O)C=Cc1ccc(c(c1)OC)O

Physicochemical Properties	
Formula	C12H14O4
Molecular weight	222.24 g/mol
Num. heavy atoms	16
Num. arom. heavy atoms	6
Fraction Csp3	0.25
Num. rotatable bonds	5
Num. H-bond acceptors	4
Num. H-bond donors	1
Molar Refractivity	60.75
TPSA	55.76 Å²

Lipophilicity	
Log $P_{o/w}$ (iLOGP)	2.76
Log $P_{o/w}$ (XLOGP3)	2.20
Log $P_{o/w}$ (WLOGP)	1.87
Log $P_{o/w}$ (MLOGP)	1.58
Log $P_{o/w}$ (SILICOS-IT)	2.17
Consensus Log $P_{o/w}$	2.11

Water Solubility	
Log *S* (ESOL)	-2.55
Solubility	6.24e-01 mg/ml ; 2.81e-03 mol/l
Class	Soluble
Log *S* (Ali)	-3.01
Solubility	2.20e-01 mg/ml ; 9.88e-04 mol/l
Class	Soluble
Log *S* (SILICOS-IT)	-2.53
Solubility	6.57e-01 mg/ml ; 2.96e-03 mol/l
Class	Soluble

Pharmacokinetics	
GI absorption	High
BBB permeant	Yes
P-gp substrate	No
CYP1A2 inhibitor	Yes
CYP2C19 inhibitor	No
CYP2C9 inhibitor	No
CYP2D6 inhibitor	No
CYP3A4 inhibitor	No
Log K_p (skin permeation)	-6.09 cm/s

Druglikeness	
Lipinski	Yes; 0 violation
Ghose	Yes
Veber	Yes
Egan	Yes
Muegge	Yes
Bioavailability Score	0.55

Medicinal Chemistry	
PAINS	0 alert
Brenk	1 alert: michael_acceptor_1
Leadlikeness	No; 1 violation: MW<250
Synthetic accessibility	2.31

Supplementary Table S2 | The prediction on brain-blood barrier (BBB) permeability of EF by Swiss ADME software. BBB permeant 'Yes' referred in the table represented

that EF can permeate through BBB.

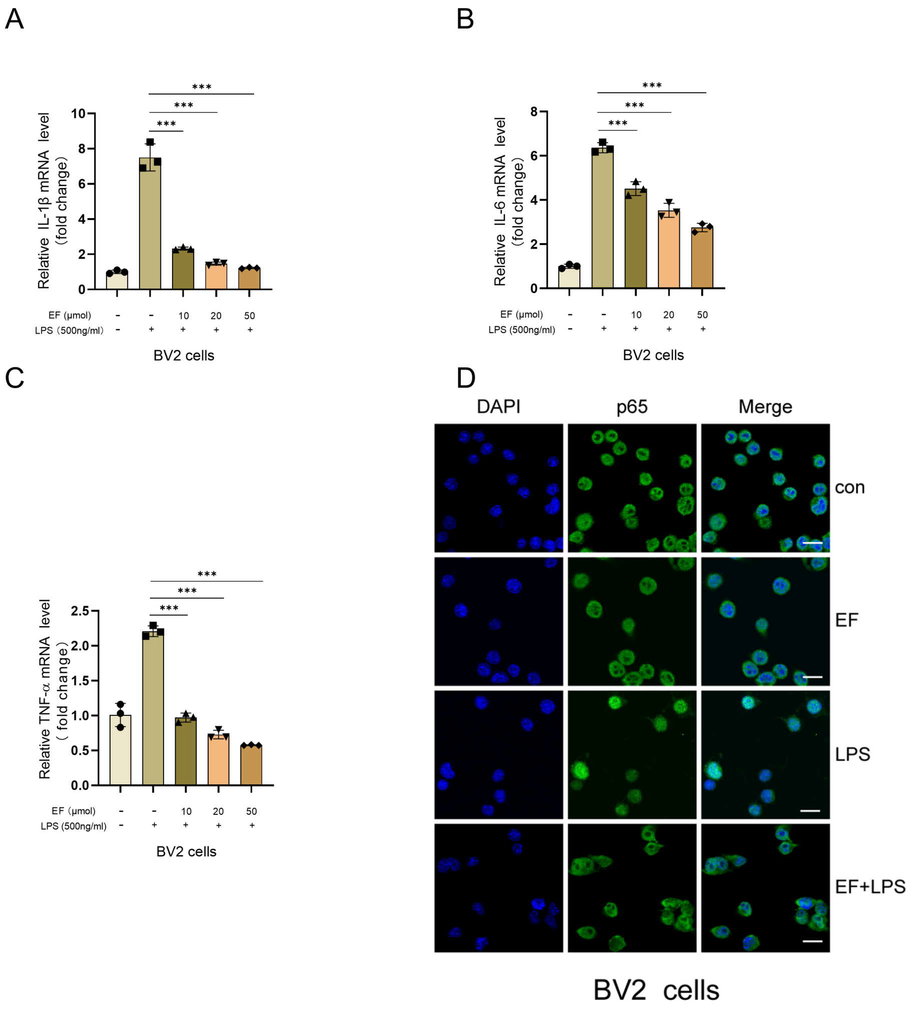

Supplementary Figure S1 | EF inhibited the expressions of pro-inflammatory factors in LPS-treated BV2 cells. (**A-C**) BV2 cells were treated with EF or not in a dose-dependent manner (10, 20, 50μmol) for 2h, followed with LPS (500ng/ml) stimulation for additional 3h. Total RNA was extracted and the mRNA levels of IL-1β (**A**), IL-6 (**B**) and TNF-α (**C**) were analyzed by RT-qPCR. Values are shown as means ± SEM. ***$p<0.005$ compared with LPS-treated BV2 cells. (**D**) BV2 cells were pre-treated with EF (50μmol) or not, then stimulated with LPS (500ng/ml) for 1.5h, followed by staining with NF-κB p65 (green) and DAPI (blue). Immunofluorescence microscopy was used to observed the localization of NF-κB and nuclei. Scale bars=20μm. The values are shown as the mean ± SEM, n=3/group. ***$p<0.05$ compared with LPS-treated groups. N.d represented none-detection. Scale bars=20μm. Comparison was from at least three independent experiments.

Target-prediction of EF in human (Top 10)					
Target	Common name	Uniprot ID	ChEMBL ID	Target Class	Probabiliy *
Monoamine oxidase B	MAOB	P27338	CHEMBL2039	Oxidoreductase	0.98024384
Carbonic anhydrase IX	CA9	Q16790	CHEMBL3594	Lyase	0.26829496
Carbonic anhydrase II	CA2	P00918	CHEMBL205	Lyase	0.19915987
Carbonic anhydrase VII	CA7	P43166	CHEMBL2326	Lyase	0.19915987
Carbonic anhydrase I	CA1	P00915	CHEMBL261	Lyase	0.19915987
Carbonic anhydrase XII	CA12	O43570	CHEMBL3242	Lyase	0.19915987
Carbonic anhydrase XIV	CA14	Q9ULX7	CHEMBL3510	Lyase	0.19915987
Carbonic anhydrase VA	CA5A	P35218	CHEMBL4789	Lyase	0.1210968
Carbonic anhydrase VI	CA6	P23280	CHEMBL3025	Lyase	0.10376176
Carbonic anhydrase XIII	CA13	Q8N1Q1	CHEMBL3912	Lyase	0.10376176

Target-prediction of EF in mouse (Top 10)					
Target	Common name	Uniprot ID	ChEMBL ID	Target Class	Probabiliy *
Monoamine oxidase B	Maob	Q8BW 75	CHEMBL3050	Enzyme	0.980244
Carbonic anhydrase VII	Ca7	Q9ERQ 8	CHEMBL2216	Enzyme	0.19916
Carbonic anhydrase XIII	Ca13	Q9D6N 1	CHEMBL2186	Enzyme	0.103762
Matrix metalloprotein ase-2	Mmp2	P33434	CHEMBL3095	Protease	0.086443
Protein tyrosine phosphatase 1B	Ptpn1	P35821	CHEMBL3336	Phosphata se	0.077758
Arachidonate 5-lipoxygenase	Alox5	P48999	CHEMBL5211	Oxidoredu ctase	0.077758
Toll-like receptor 4	Tlr4	Q9QU K6	CHEMBL17951 67	Toll-like and Il-1 receptors	0.069097
Epidermal growth factor receptor erbB1	Egfr	Q01279	CHEMBL3608	Kinase	0.069097
Adenosine A2b receptor	Adora2b	Q60614	CHEMBL2237	Family A G protein-coupled receptor	0.069097
Adenosine A1 receptor	Adora1	Q60612	CHEMBL3688	Family A G protein-coupled receptor	0.060425

Supplementary Table S3 | Predictions on MAO-B showing as a target of EF both in human and mouse by SwissTargetPrediction website. Top 10 targets were listed in the table. MAO-B both in human and mouse shows a higher probability in binding to EF.

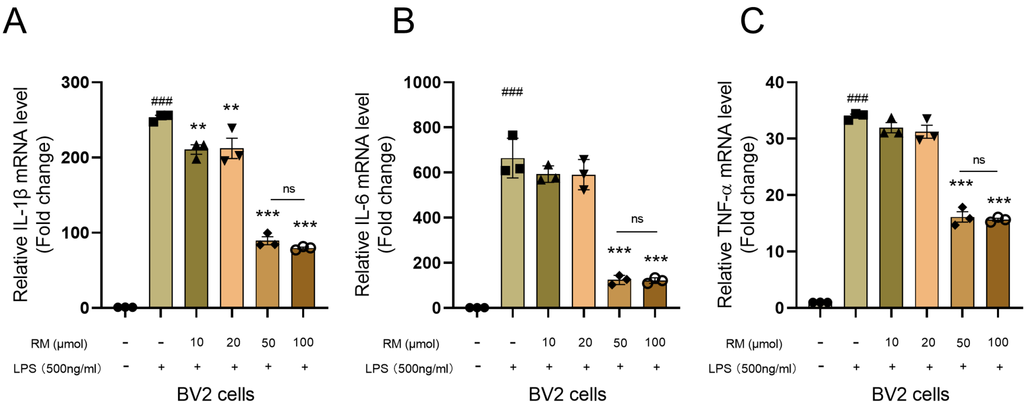

Supplementary Figure S2 | MAO-inhibitor RM inhibited the expressions of pro-inflammatory in LPS-treated BV2 cells. (**A-C**) BV2 cells were treated with EF or not as above for 2h, followed with LPS (500ng/ml) stimulation for additional 3h. Total RNA was extracted and the mRNA levels of IL-1β (**A**), IL-6 (**B**) and TNF-α (**C**) were analyzed by RT-qPCR. Values are shown as means ± SEM. ###p<0.005 versus control. **p<0.05, ***p<0.005 compared with LPS-treated groups.

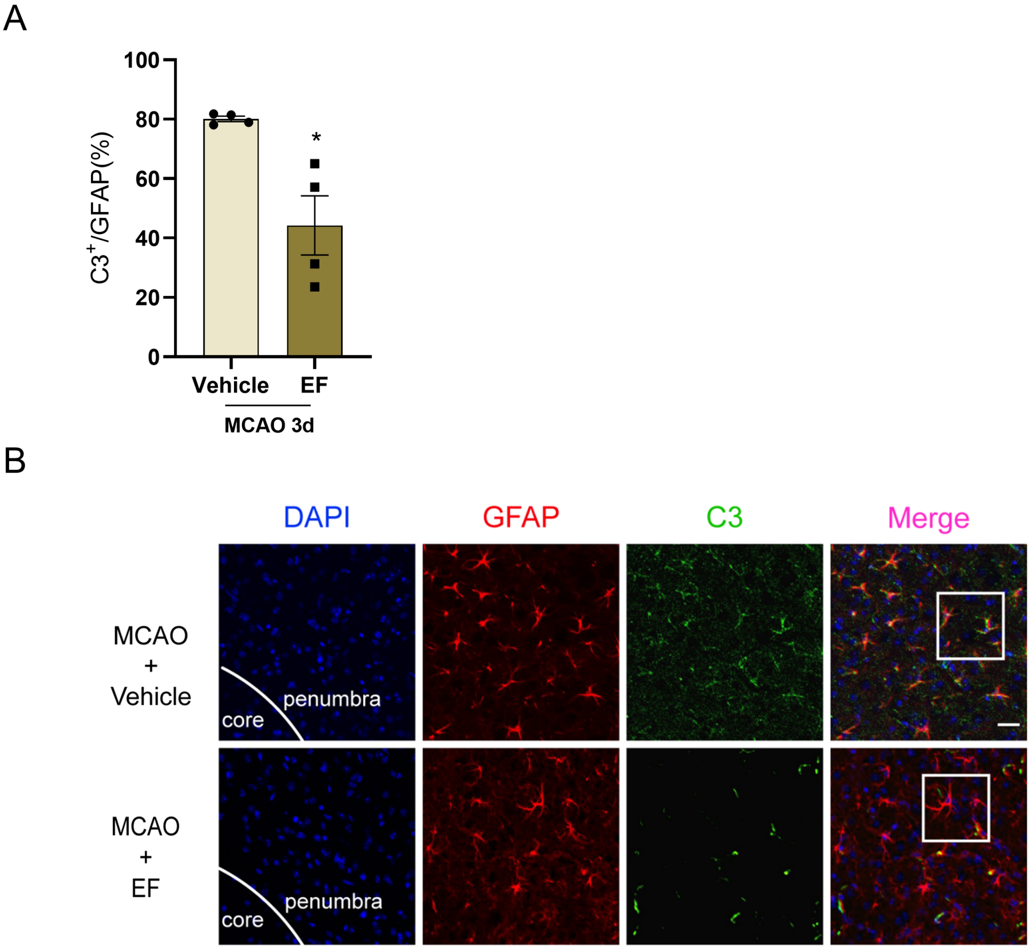

Supplementary Figure S3 | EF inhibited the astrocytes reaction secondary to

activated-microglia after ischemia. (**A**) The percentage of C3-positive GFAP astrocytes in the ischemic penumbra of MCAO mice with/without EF injection was counted by ImageJ. (**B**) Representative images of brain tissue sections were obtained from tMCAO mice treated with/without EF, staining with C3 (green), GFAP (red) and DAPI (blue). scale bar=50μm.Values are shown as means ± SEM, n=4/group. * $p<0.5$ compared with vehicle-treated MCAO mice.

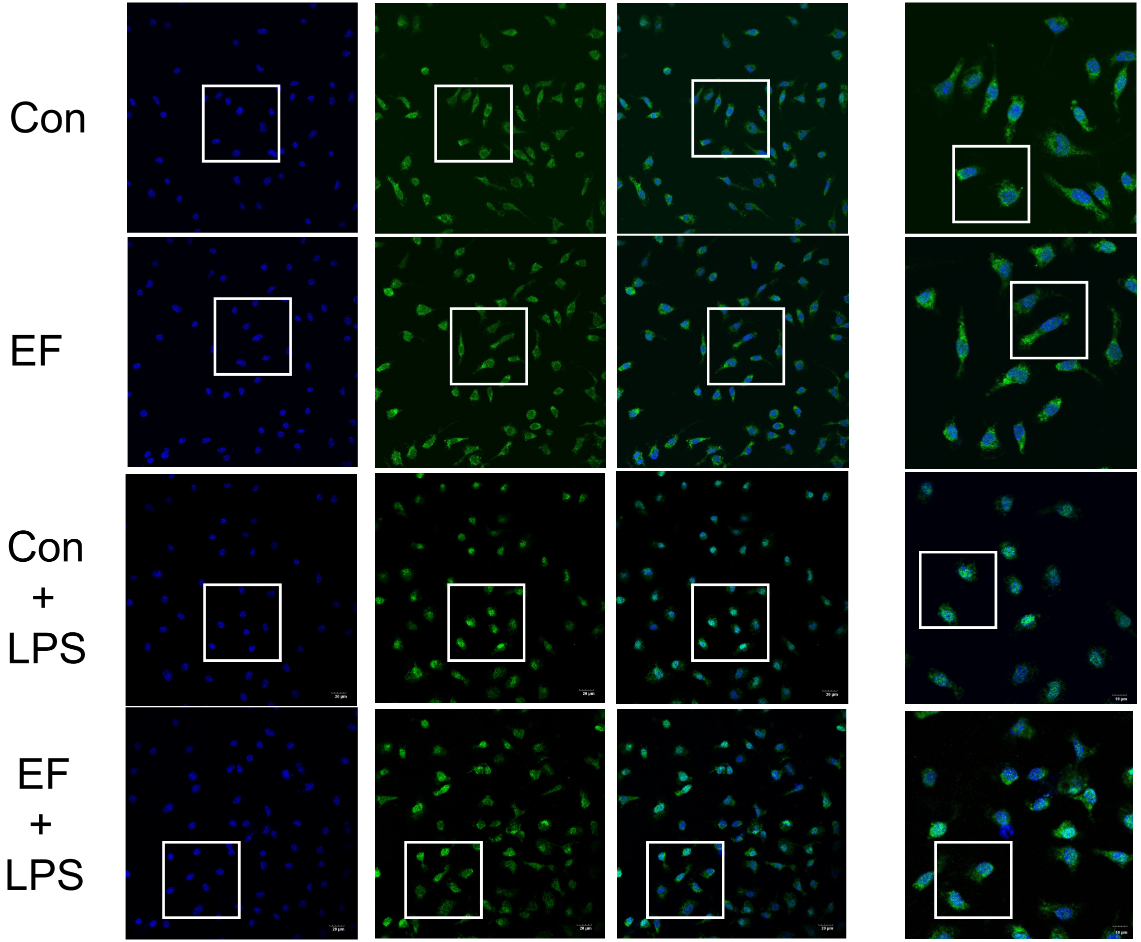

Supplementary Figure S1| IF image of NF-κB in LPS-induced primary microglia with/without EF treatment. (In Figure 2N)

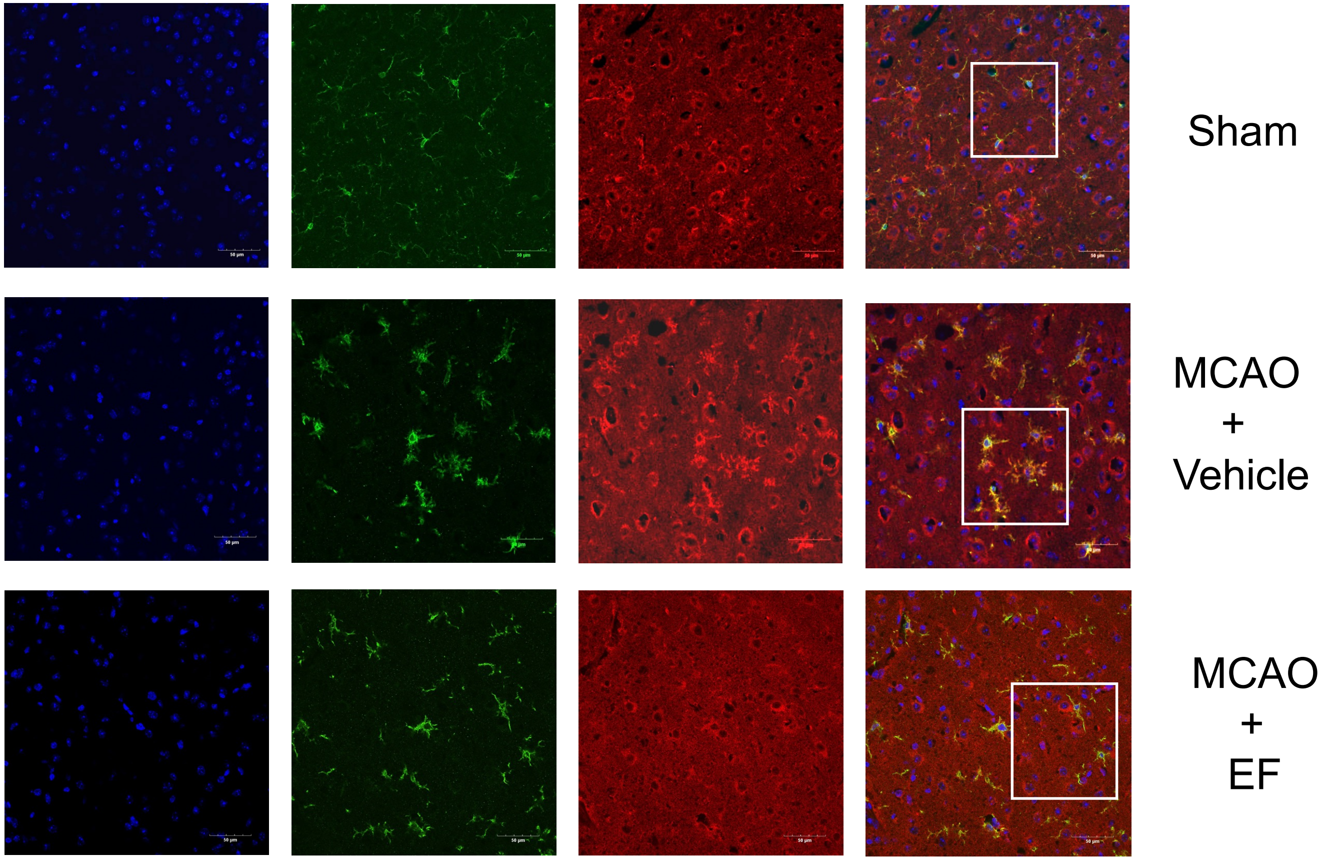

Supplementary Figure S2| IF image of TNF-α in MCAO 3day mice with/without EF treatment. (In Figure 3 G)

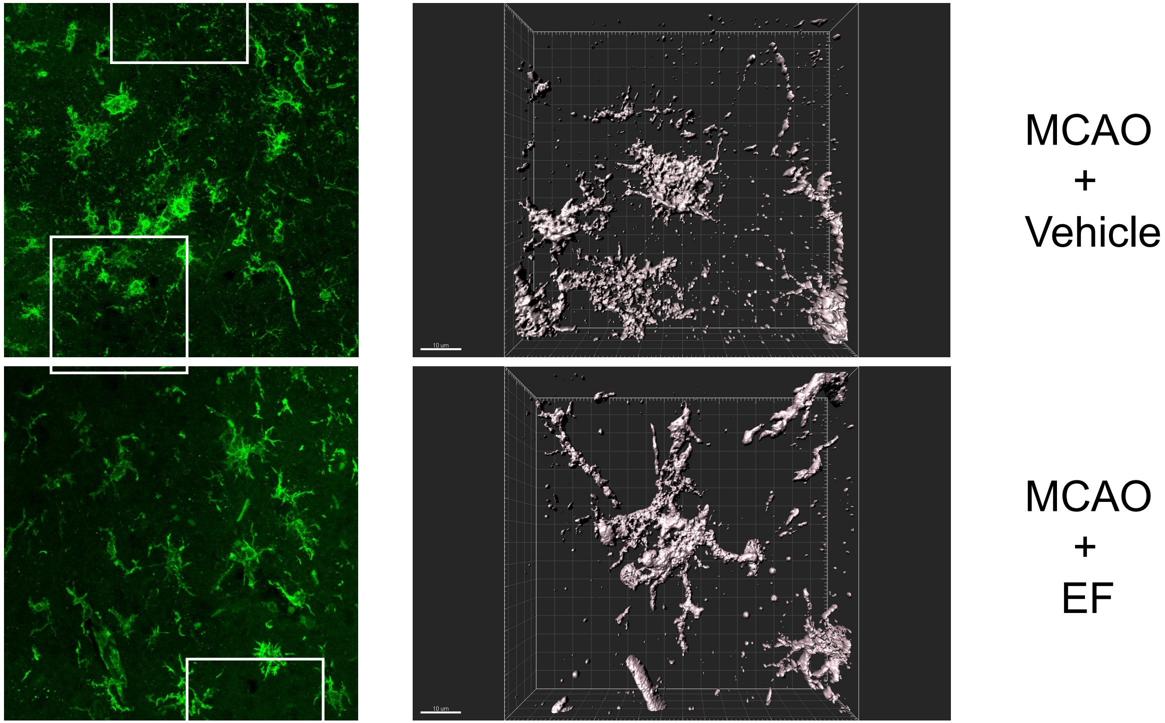

Supplementary Figure S3| IF image of microglial morphology in mice brain slices at MCAO 3day with/without EF treatment. (In Figure 3 H)

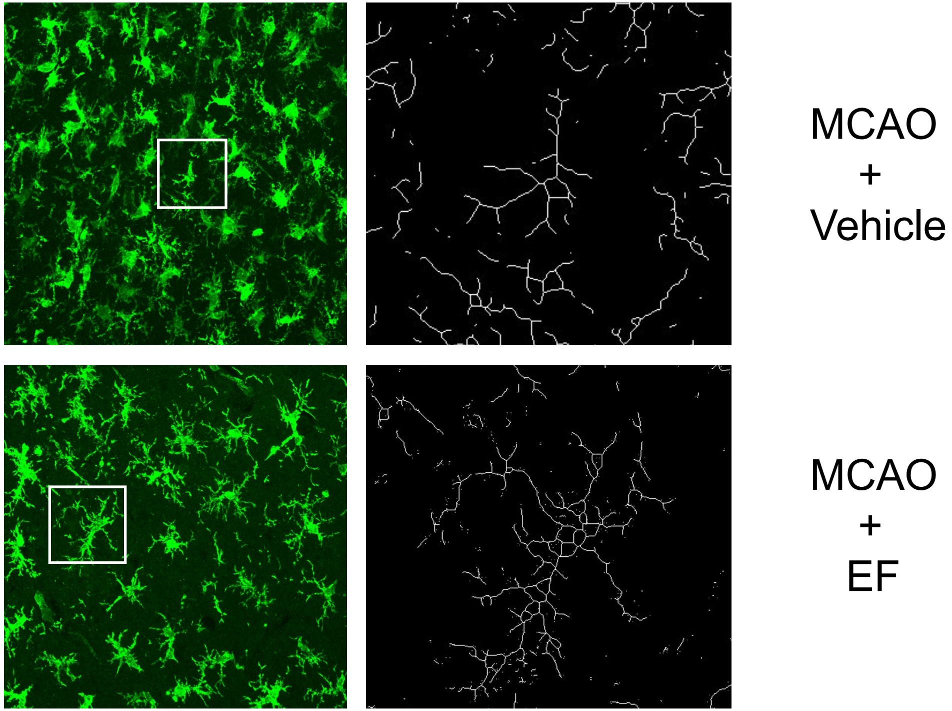

Supplementary Figure S3| IF images with corresponding binary of microglial morphology in mice brain slices at MCAO 3day with/without EF treatment. (In Figure 3 I)

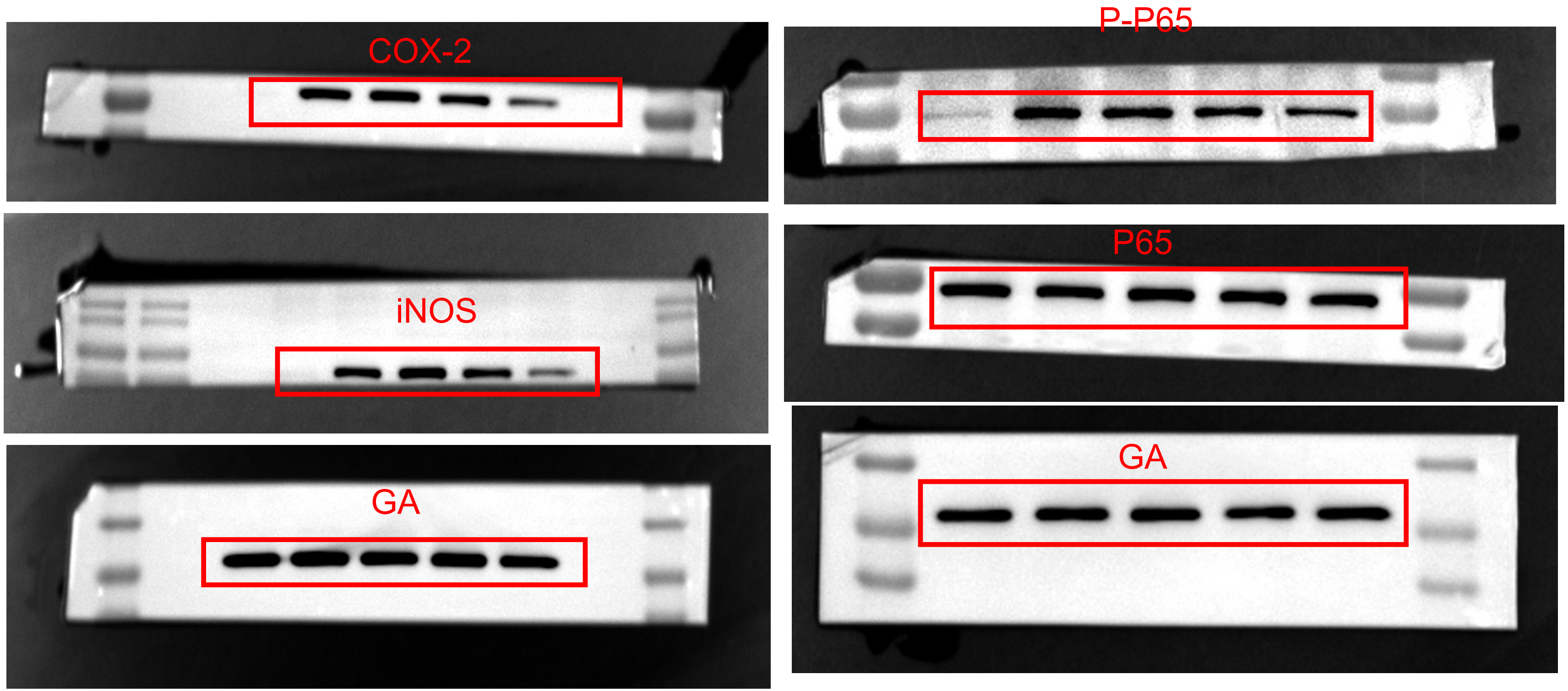

Supplementary Figure S5| WB images of COX-2, iNOS, GAPDH, P-P65, P65 in LPS-induced primary microglia with/without EF treatment. (In Figure 2J-M)

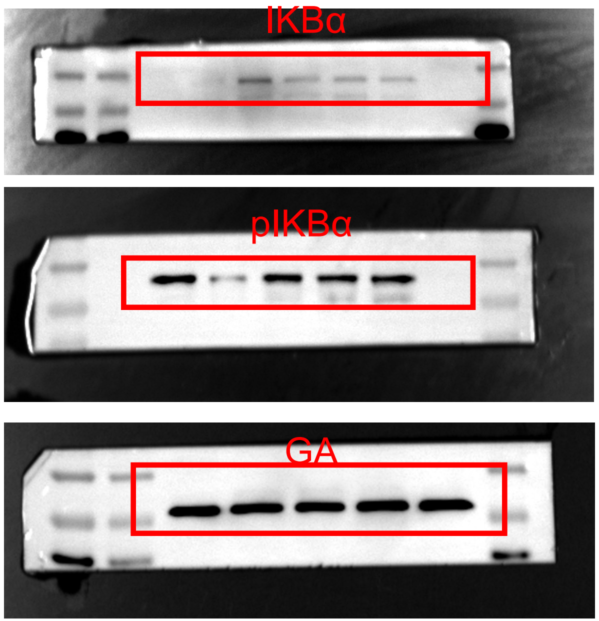

Supplementary Figure S6| WB images of pIκBα, IκBα, and GAPDH in LPS-induced primary microglia with/without EF treatment. (In Figure 2M)

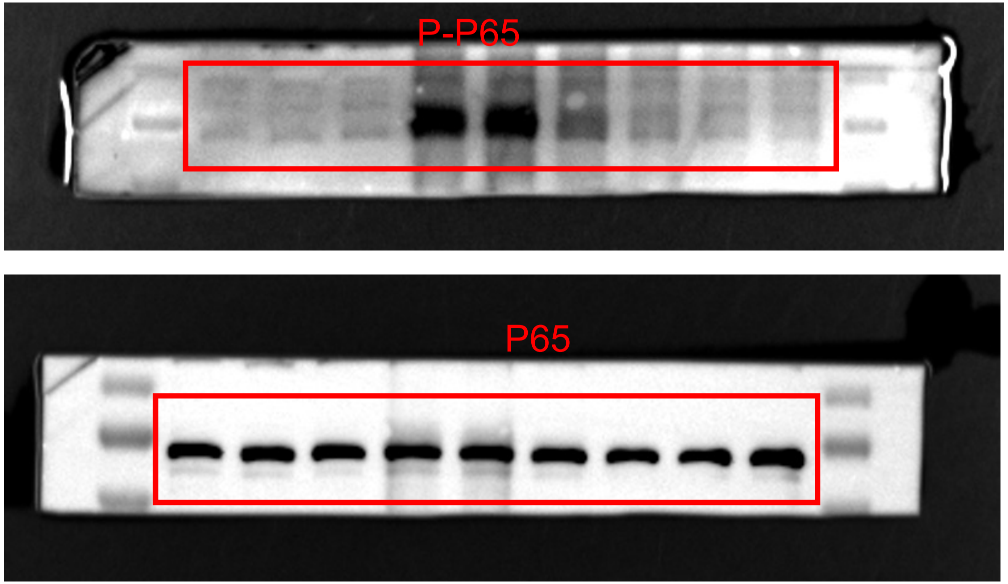

Supplementary Figure S7| WB images of P-P65, P65 of grouped mice brain tissue with/without EF treatment in MCAO 3day. (In Figure 3E)

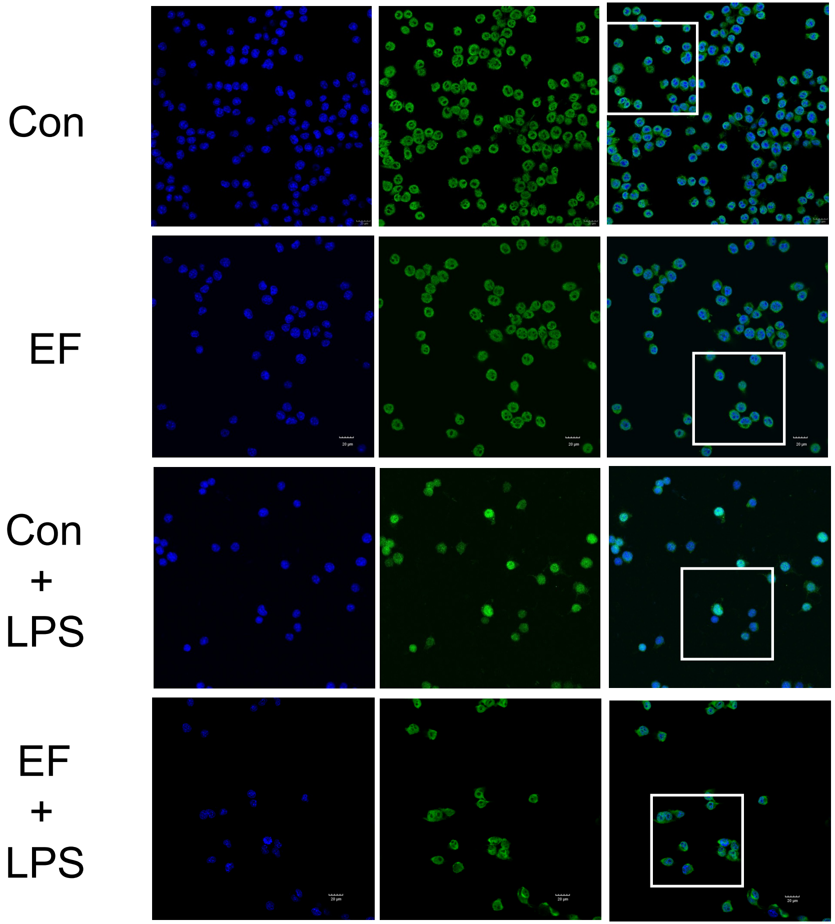

Supplementary Figure S8 | IF image of NF-κB in LPS-induced BV2 cells with/without EF treatment. (In Supplementary Figure S2)

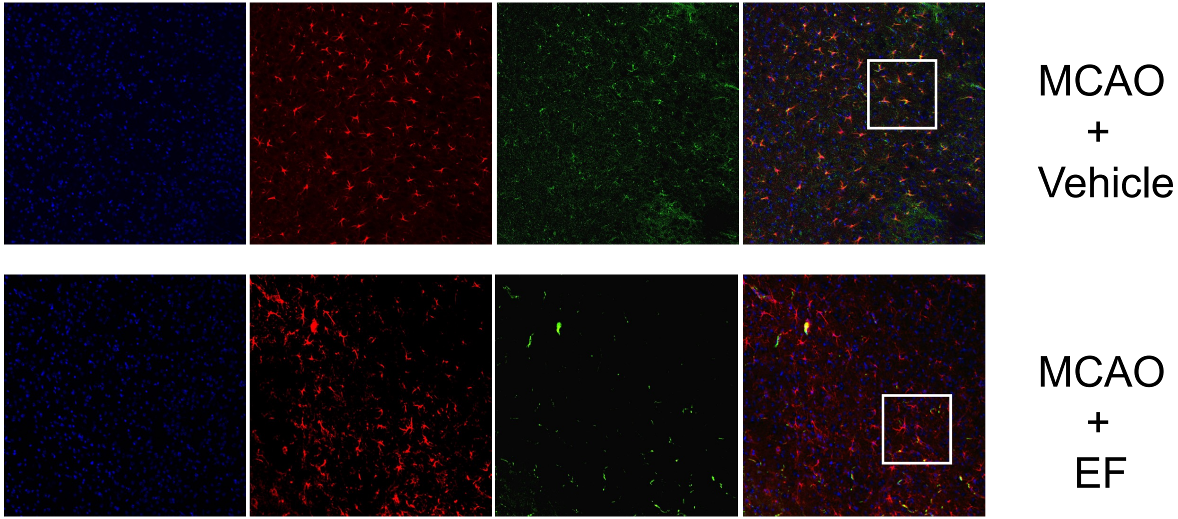

Supplementary Figure S9 | IF image of C3+ GAFP astrocytes in mice brain slices at MCAO 3day with/without EF treatment. (In Supplementary Figure S3)
